# Sampling investigation and statistical analysis for mental problems of freshmen in Shandong Province

**DOI:** 10.1038/s41598-022-25114-4

**Published:** 2022-11-29

**Authors:** Chang Li, Bingchuan Sun

**Affiliations:** 1 College of Insurance, Shandong University of Finance and Economics, 40 Shungeng Road, Jinan, China; 2 College of Physical Education, Shandong University of Finance and Economics, 40 Shungeng Road, Jinan, China

**Keywords:** Applied mathematics, Statistics

## Abstract

niversity stage, especially the freshmen stage, is a high incidence stage of students’ psychological problems. Effective sampling investigation and statistical analysis of freshmen’s mental health problems are conducive to solve freshmen’s problems and prevent further crises. In the past 4 years (2018–2021), using Chinese college students’ mental health screening scale, we have taken probability proportionate to size sampling investigations about the mental problems to 9882 freshmen in 45 public universities in Shandong Province near the end of their first semester. Based on these data, we conducted the comparison of anxiety and depression for post pandemic era vs. pre pandemic era by analysis of variance, and analysed the influencing factors for anxiety and depression by linear regression and canonical correlation analysis. The results indicate the extents of anxiety and depression in post pandemic era are significantly more severe than pre pandemic era. Inferiority complex, obsession and somatization are the main effect factors of anxiety and depression. To the best of our knowledge, this research is the first systematical investigation and analysis for the mental problems among freshmen in the whole Shandong Province before and after the epidemic. The research results are conducive for the mental health counseling and intervention of freshmen’s mental problems, and also helpful for policy making and prevention for psychological crisis management.

## Introduction

The mental health of college students, especially for the freshmen, is an area of increasing concern worldwide. Previous studies suggest high rates of psychological morbidity, especially depression and anxiety, among university or college students^1–3^. Adlaf et al.^4^ found a remarkable inverse relationship between years of study and mental health in university students, that is, the freshmen are at greatest risk on mental health. Other related typical researches include^5^: conducted a web-based survey of depression, anxiety and stress for freshmen in Hong Kong, and found the high prevalence of anxiety and depression symptoms in freshmen is alarming. Cheung et al.^6^ conducted a cross-sectional survey study for depression and anxiety to freshmen in a university in Hong Kong. Wei^7^ investigated the prevalence of mental health problems and their predictors for Chinese college freshmen in Shanghai.

In December 2019, the coronavirus disease 2019 (COVID-19) outbreak occurred. The pandemic and consequent compulsory measures in China, such as quarantine, lockdown and containment measures of communities and universities, have great impact on people’s normal lives and mentality, and give birth to a new term: post pandemic era (or post COVID-19 era). In the post pandemic era, the economic situation, employment, government management style have undergone great changes, and all of them have significant impacts on people’s mentality health. These lockdown measures causes unprecedented pressure and the surge in mental problems^8^. Thus there is a tremendous need to compare the mental problems of students between post pandemic era and pre pandemic era. Shi et al.^9^ conducted a large-sample, cross-sectional online survey for the prevalence of and risk factors associated with mental health symptoms in China during the COVID-19 era. Vadivel et al.^10^ proposed the mental health and coping strategies in undergraduate students during the pandemic. Some other typical researches about mental health problems related to COVID-19 pandemic include:^11–13^, etc.

In China, the first COVID-19 patient was found in December 2019, and the consequent compulsory measures, especially the lockdown and containment measures of universities and communities, began at 2020. Thus the post pandemic era in China is usually considered to start at 2020.

Previous works have great theoretical and practical significance in the research of mental health problems for freshmen, and provide solid foundations for the future research. However, the thorough studies for the mental problems of freshmen in some typical provinces before and after COVID-19 pandemic, and the comparison of the extent of anxiety and depression of freshmen between pre pandemic era and post pandemic era as well as the psychological effect factors for anxiety and depression, are seldom examined in previous researches.

To analyse the extent of the depression and anxiety of freshmen between post pandemic era and pre pandemic era, and the psychological effect factors of depression and anxiety in recent years, we took sampling investigation and statistical analysis for mental problems, especially for anxiety and depression, of freshmen in Shandong Province in the past 4 years (2018–2021). These results are conducive for the mental health counseling and intervention of freshmen’s mental problems, and helpful for promoting psychological health for freshmen.

The reasons that we choose Shandong Province as the research object are: the numbers of universities and undergraduates of Shandong Province rank first in northern China, and second in the whole China. Thus it is the most representative province for northern China. Besides, Shandong Province was one of the most severely affected provinces in the COVID-19 pandemic, and the compulsory measures in this province was one of the strictest in China. In a large number of control areas in Shandong Province, people are required not to leave the universities or communities unless absolutely necessary. Thus it is suitable for the research of the effect of COVID-19 and related compulsory measures on mental problems. The mental health problem of college students in Shandong Province in post pandemic era have triggered intense attention in the public and academia. Recent typical research in this area include^14^: assessed the mental health problems for nonmedical vs. medical college students in universities of Shandong Province during the COVID-19 epidemic. Su et al.^15^ proposed a preliminary study on the mental health status and influencing factors of college students in Shandong University of Traditional Chinese Medicine during the COVID-19 pandemic era. Zhang^16^ conducted a online survey for the anxiety of students in a university in Shandong Province during the COVID-19 era.

## Study design and sampling investigation method

### Study design

In the past 4 years, we have taken a large sample investigations to freshmen in 45 public universities in Shandong Province near the end of their first semester. This was an anonymous survey, and confidentiality of data was ensured. The main scale of the investigations is the Chinese college students’ mental health screening scale made by the Ministry of Education of China (http://xinli.gzedu.com/dist/index.html#/evaluation/login, https://www.wjx.cn/xz/151046982.aspx). Except for participants’ demographic characteristics, this scale includes 22 items related to psychological problems: anxiety, depression, bigotry, inferiority, sensitive, social phobia, somatization, dependency, hostile attack, impulsion, obsession, internet addiction, self injurious behavior, eating problems, sleep disturbance, university adaptation difficulties, interpersonal troubles, academic pressure, employment pressure, trouble in courtship, suicidal intent, hallucinations and delusions. The score of each item is index standard score (Z-score), which is proportional to the severity of the mental problem. The reliability and validity indexes of the scale was provided in detail in^17^. Based on these data, we conducted the comparison of the extent of anxiety and depression for pre pandemic era vs. post pandemic era and analyzed the mental effect factors for them in Section “Data analysis methods” and “Results”.

Since in China, the COVID-19 related questions (such as the lockdown and containment measure of universities and communities and their influence) are sensitive and strictly restricted in questionnaires, our questionnaires did not have questions about impact of the COVID-19 directly.

### Sampling investigation method

The principles and methods of sampling investigation have been introduced in^18–20^, etc. By these principles, we formulated the sampling investigation method and sample size.

Our sampling investigation adopted PPS (probability proportional to the population size sampling). In year 2018–2021, we randomly chose the freshmen in the 45 public universities in Shandong Province near the end of their first semester by probability sampling, and sent the scales of the investigations to them by Enterprise WeChat of each university. The sample size in each university is proportional to the number of freshmen in this university. The age of the freshmen usually range from 18 to 20. All investigation protocols were approved by the Research Ethics Committee of Shandong University of Finance and Economics in China, and conducted in compliance with the American Association for Public Opinion Research (AAPOR) reporting guideline. Written informed consents were obtained online from all participants and/or their legal guardians before the they began to fill in the questionnaire. According to the confidentiality agreement with the respondent, the information of students and institutions are confidential.

### Determination of sample size

In PPS sampling, the minimum sample size is usually computed by the formula^21^:

1 $$\begin{aligned} N=\frac{u_\alpha ^2p(1-p)}{d^2} \end{aligned}$$

Where N is the minimum sample size under a certain confidence level and the error rate. This investigation adopts 95 percent confidence level, which is most frequently used in similar investigations. Thus $u_\alpha =1.96$. Since the *p* value is difficult to estimate, we adopt a conservative strategy and take $p=0.5$. To guarantee the accuracy of the investigation, we set the error rate (maximum allowable absolute error) to 2 percent. Thus the minimum sample size should be: $\frac{1.96^2\times 0.5\times 0.5}{0.02^2}=2401$ for each grade.

According to national census regulations promulgated by the State Council of China (http://www.gov.cn/zhengce/2020-12/27/content_5574683.htm), the proportion of population sampling survey should no less than 1 percent of the total number of people. The number of freshmen in public universities in Shandong Province in past 4 years is in Table 1 (According to the record convention in Chinese universities, each student’s grade is the year of entrance of this student in the university). Thus on each grade, around 3000 students were chosen by PPS sampling in the 45 public universities.

**Table 1:** Total number of undergraduate students in each grade.

Grade	Total number
Grade of 2018	272078
Grade of 2019	279085
Grade of 2020	286766
Grade of 2021	294373

## Data analysis methods

We used SAS 9.4 and R 4.1.2 for data analysis. Descriptive statistics were used to present demographic data.

Conducting statistical analysis for all items is a huge work. By a certain number of former research results, such as^7, 13, 22, 23^, anxiety and depression are the most common and representative mental health problems affecting freshmen. We have also confirmed these results through questionnaires and interviews with a certain number of students. Thus, we take anxiety and depression as the two main indicators of mental problems for freshmen, and mainly focus on the analysis of these two items in this research. Other items will be studied in detail in the future work.

### Comparison of the extent of anxiety and depression in pre pandemic era vs. post pandemic era

We used ANOVA ( analysis of variance) to compare the extent of anxiety and depression in pre and post COVID-19 pandemic era. What we were interested in is the contrast of anxiety and depression of pre pandemic era vs. post pandemic era, thus we made a contrast for anxiety and depression between 2 groups: grade 2018–2019 and grade 2020–2021. If the result indicated significant difference in the extent of anxiety or depression before and after COVID-19 pandemic, then we went a step further and did multiple comparison for the mean of the scores of depression in each year. To make the manuscript concise, all program results are in attachment.

### Analysis of psychological effect factors for anxiety and depression in pre pandemic era and post pandemic era

According to former studies, anxiety and depression are usually comorbid, and anxiety depression (also known as comorbid anxiety and depression) is relatively a common syndrome^24–27^. A certain number of former studies point out, anxious depression have different neurobiological profiles compared to non-anxious depression, and patients with anxious depression had more frequent episodes of major depression^24, 28–30^. Thus we took anxiety and depression as the 2 representative mental problem indicators, and considered them together as a whole.

Since some effect factors of psychological problems are high privacy and can not be reflected in the scale (such as genetic genes, family income, childhood experience, etc.), it is a huge work to study all effect factors for anxiety and depression exhaustively. Thus in this manuscript, we mainly focus on the statistical analysis for the psychological effect factors of anxiety and depression in pre pandemic era and post pandemic era.

The statistical analysis process is composed of two steps: first, we get the significant psychological effect factors of anxiety and depression by linear regression separately; second, we conduct canonical correlation analysis to these significant psychological effect factors (as the independent variables) and anxiety and depression (as the dependent variables). The core idea of canonical correlation analysis is to transform the correlation between multiple variables into the correlation between two representative variables^31^.

## Results

### Descriptive statistics for the sampling investigation

The total number of valid questionnaires collected and the number of mental problems detected are in Table 2. The mental problems are divided into three levels according to the severity in descending sequence, that is, first-level is the most serious problem.

**Table 2:** Number of valid questionnaires and mental problems detected.

Grade	Total number	First-level	Second-level	Third-level	Sum	Percentage
Grade of 2018	2502	88	199	180	467	0.1867
Grade of 2019	2455	92	213	227	439	0.1788
Grade of 2020	2484	133	131	168	432	0.1739
Grade of 2021	2441	150	245	212	607	0.2487
Total	9882	463	788	694	1945	0.1968

In Table 2, the total number is the number of valid questionnaires collected in each grade, and sum is the total number of students diagnosed with mental problems. Percentage is the percentage of students diagnosed with mental problems. From Table 2, the percentage of mental problems in year 2021 is significantly higher than other 3 years. The main reason is in year 2021, the pandemic has lasted for more than one year. With the passage of time, a growing number of students are influenced by the pandemic and related control measures, thus there is a marked increase on mental problems.

The descriptive statistics of the demographic and social characteristics of the participants, such as grade, gender and region are in Table 3. The percentage is in parentheses. The region is the student’s hometown region.

**Table 3:** Demographic and social characteristics of the participants.

Grade	Total number	Male	Female	Countryside	City
Grade of 2018	2502	1185 (47.4%)	1317 (52.6%)	1006 (40.2%)	1496 (59.8%)
Grade of 2019	2455	1098 (44.7%)	1357 (55.3%)	978 (39.8%)	1477 (60.2%)
Grade of 2020	2484	1135 (43.7%)	1349 (54.3%)	1022 (41.4%)	1462 (58.6%)
Grade of 2021	2441	1204 (49.3%)	1237 (50.7%)	967 (39.6%)	1474 (60.4%)

**Figure 1:**
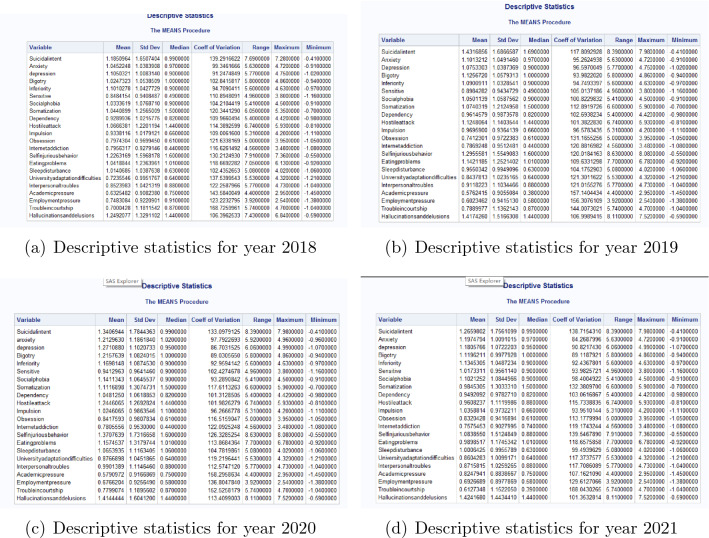
The descriptive statistics for mental problems in year 2018–2021.

The mean, standard deviation (SD) and score range of all psychological problem indicators of the participants in each year is in Fig. 1 (in appendix).

### Comparison of the extent of anxiety and depression in pre pandemic era vs. post pandemic era

**Figure 2:**
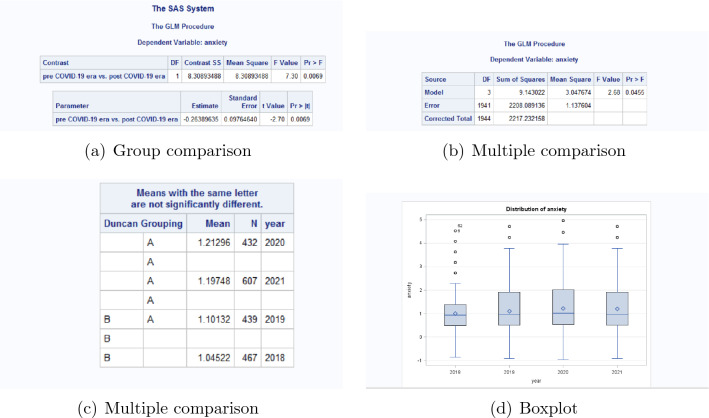
Comparison of anxiety for post pandemic era versus pre pandemic era.

The contrast of anxiety of pre pandemic era vs. post pandemic era is in Fig. 2. From the result (a) in Fig. 2, the *P* value of the contrast of anxiety of pre pandemic era vs. post pandemic era is 0.0069, which means there is significant difference between the extent of anxiety between pre pandemic era and post pandemic era. Thus we have to do multiple comparison for the means of anxiety in each year.

From the results (b) and (c) for multiple comparison in Fig. 2, the means of anxiety in year 2020 and 2021 are marked as A, the means of anxiety in year 2019 is marked as A and B, and the means of anxiety in year 2018 is marked as B. The *P* value of the multiple comparison is 0.0455. This means comparing with pre pandemic era, the extent of anxiety of post pandemic era is significantly higher. The boxplots (d) also confirm that conclusion.

**Figure 3:**
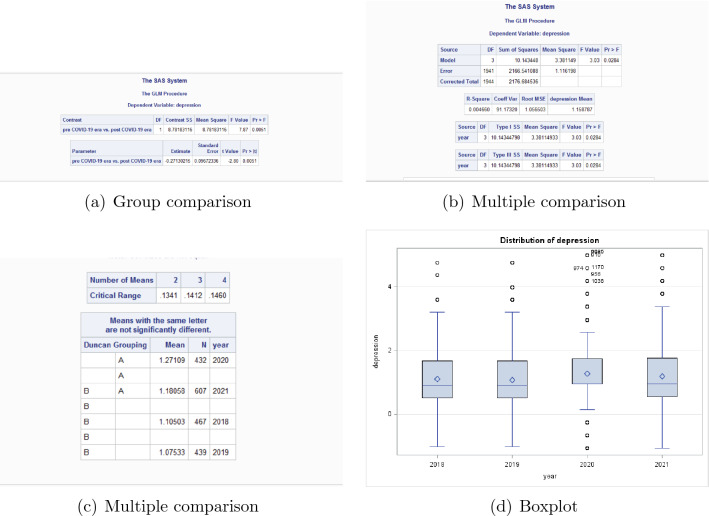
Comparison of depression for post pandemic era versus pre pandemic era.

The contrast of depression of pre pandemic era vs. post pandemic era is in Fig. 3. From Fig. 3, the *P* value of the contrast of depression of pre pandemic era vs. post pandemic era is 0.0051, which means there is significant difference between the extent of depression before and after COVID-19 pandemic. Thus we have to do multiple comparison for the mean of the scores of depression in each year.

From the results for multiple comparison, the mean of depression in year 2020 is marked as A, the mean of depression in year 2021 is marked as A and B, and the means of depression in year 2018 and 2019 are marked as B. The *P* value of the multiple comparison is 0.0284. This means comparing with pre pandemic era, the extent of depression in post pandemic era is significantly higher. The boxplots also confirm that conclusion.

From Figs. 2 and 3, the extents of both anxiety and depression in 2020 are the highest among the 4 years. The main reason is the lockdown and containment measures of universities and even the whole society in Shandong Province is the most severe in year 2020, which caused enormous disruptions to daily life. These measures are unprecedented in most freshmen’s life, thus the psychological discomfort, including anxiety and depression is significantly more severe. On the other side, from Table 2, the percentage of mental problems in year 2021 is the highest among the 4 years.

These results indicate comparing with pre pandemic era, the extent of anxiety and depression of freshmen are significantly more severe in post pandemic era.

Lu Lin, the academician of the Chinese Academy of Science, pointed out the pandemic have severe effect on people’s mental health, especially on anxiety and depression, and nearly one third of the isolated residents suffer from depression, anxiety and insomnia (https://weibo.com/ttarticle/p/show?id=2309634809997371703510). Other typical research, such as^32, 33^, also had similar viewpoint. On the other side, the pandemic and compulsory measures in Shandong province was one of the most severe in China. In a large number of control areas in Shandong Province, college students were isolated in universities or communities, or even in a dormitory building for a long time, and were not permitted to leave unless absolutely necessary.

Based on these results and former research, the aggravation of anxiety and depression is highly correlated to the pandemic and related lockdown and containment measures. Thus psychological counseling office and other relevant departments in each university should strengthen the mental health education and instruction to freshmen in post pandemic era, pay attention to the mental health status of isolated students timely, and conduct psychological releasing and nursing for students with psychological problems to prevent further mental crises.

### The psychological effect factors for anxiety and depression in pre pandemic era and post pandemic era

**Figure 4:**
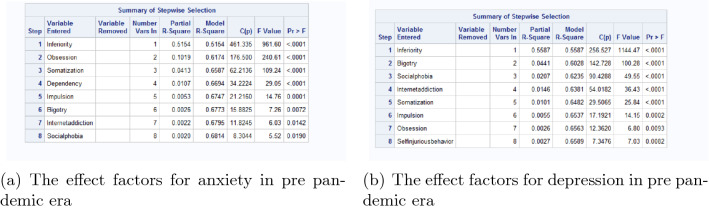
The effect factors for anxiety and depression in pre pandemic era.

**Figure 5:**
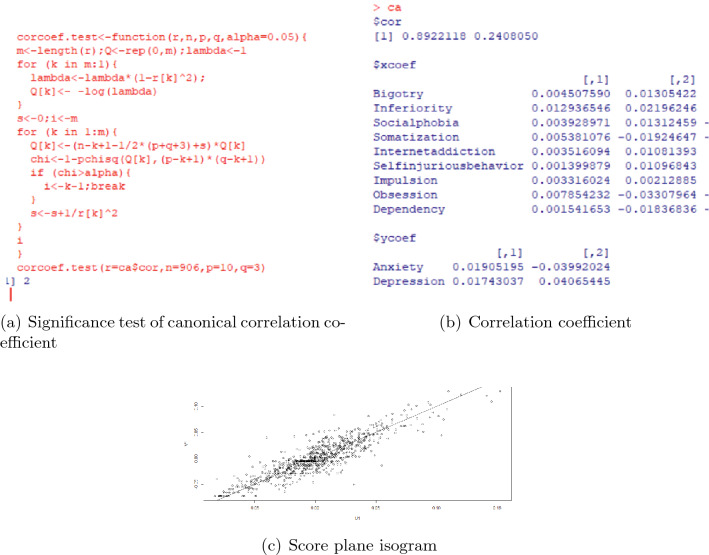
The canonical correlation analysis for anxiety and depression in pre pandemic era.

We take linear regression with stepwise selection for anxiety and depression separately to get the significant effect factors for them. The results of linear regression in pre pandemic era are in Fig. 4. Based on these significant effect factors, the canonical correlation analysis is processed, and the results are in Fig. 5. By the significance test of canonical correlation coefficient in (a), we should examine the first 2 pairs of canonical variables. However, since the correlation coefficient of the first pair of is 0.8922118 (in (b)), and the second pair is 0.2866405. Thus we only have to analyse the first pair.

Since some effect factors of psychological problems are high privacy and can not be reflected in the scale, the first pair of canonical variables with correlation coefficient 0.8922118 can be considered as highly correlated.

Denote the effect factors (xcoef) in (b) of Fig. 5 in sequence as $x_1$ through $x_9$, and the 2 dependent variables are anxiety($y_1$), and depression($y_2$). The expression of the first pair of canonical correlation variables is:

2 $$\begin{aligned} \begin{aligned} \left\{ \begin{array}{ll} &{}U= 0.004507590x_1+ 0.012936546x_2+ 0.003928971x_3+ 0.005381076x_4+0.003516094x_5\\ &{}0.001399879x_6+ 0.003316024x_7+0.007854232x_8+ 0.001541653x_9\\ &{}V= 0.01905195y_1+0.01743037y_2 \end{array} \right. \end{aligned} \end{aligned}$$

Where U is the linear combination of the effect factors (independent variables), and V is the linear combination of anxiety and depression (dependent variables).

By^31^ and^34^, The coefficient (also known as loading) in front of each component indicates the importance of this component. From this expression, the effect factors with relatively larger loadings in sequence are $x_2$, $x_8$ and $x_4$, which means inferiority complex, obsession and somatization are main effect factors to anxiety and depression. The loading of $x_1$, $x_3$, $x_5$ and $x_7$ are moderate, which means bigotry, social phobia, internet addiction and impulsion are also significant effect factors to anxiety and depression.

From the score plane isogram for the canonical correlation analysis in (c) of Fig. 5, the scatters are approximately distributed on a straight line, which means the correlation of the first pair of canonical correlation variables can be explained quite well by canonical correlation analysis, the correlation of the first pair of canonical correlation variables is stable.

**Figure 6:**
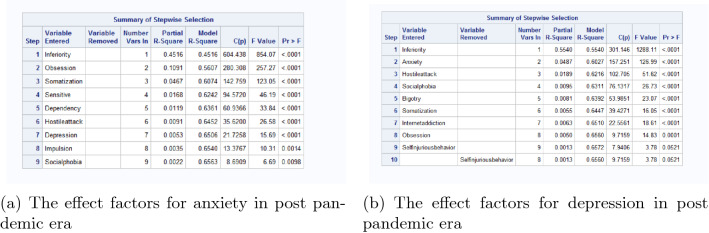
The effect factors for anxiety and depression in post pandemic era.

**Figure 7:**
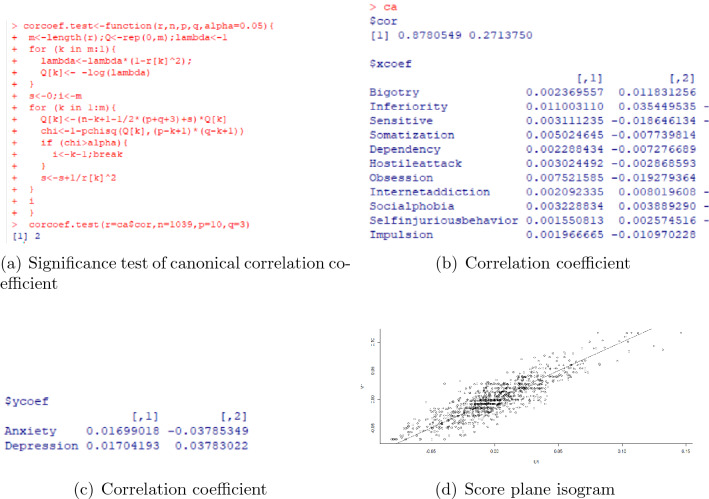
The canonical correlation analysis for anxiety and depression in post pandemic era.

The results of significant effect factors for anxiety and depression in post pandemic era are in Fig. 6. Based on these significant influencing factors, the canonical correlation analysis is processed, and the results are in Fig. 7. The correlation coefficient of the first pair of is 0.8780549, which can be considered as highly correlated, and the second pair is 0.2713750. Thus combing the results of significance test and correlation coefficient, we only have to analyse the first pair of canonical variable.

Denote the effect factors (xcoef) in (b) of Fig. 7 in sequence as $x_1$ through $x_{11}$, the expression of the first pair of canonical correlation variables is:

3 $$\begin{aligned} \begin{aligned} \left\{ \begin{array}{ll} &{}U= 0.002369557x_1+ 0.011003110x_2+ 0.003111235x_3+ 0.005024645x_4+0.002288434x_5\\ &{}+ 0.003024492x_6+0.007521585x_7+ 0.002092335x_8+0.003228834x_9+ 0.001550813x_{10}\\ &{}+ 0.001966665x_{11}\\ &{}V= 0.01699018y_1+0.01704193y_2 \end{array} \right. \end{aligned} \end{aligned}$$

From this equation, the effect factors with relatively larger loadings in order are $x_2$, $x_7$ and $x_4$, which means inferiority complex, obsession and somatization are main effect factors to anxiety and depression. The loading of $x_3$ $x_6$ and $x_9$ are moderate, which means sensitive, hostile attack and social phobia are also significant effect factors to anxiety and depression.

From the score plane isogram for the canonical correlation analysis in (c) of Fig. 7, the scatters are approximately distributed on a straight line, which means the correlation of the first pair of canonical correlation variables can be explained quite well by canonical correlation analysis, the correlation of the first pair of canonical correlation variables is stable.

Comparing the results of linear regression in Figs. 4 and 6, in post pandemic era, anxiety and depression are significant effect factors of each other. This means in post pandemic era, anxiety and depression are comorbid, and have greater mutual interactive influence to each other than pre pandemic era. In both pre pandemic era and post pandemic era, inferiority complex, obsession and somatization are the top 3 effect factors for anxiety and depression. Comparing with pre pandemic era, post pandemic era have 2 characteristic significant effect factors: sensitive and hostile attack. These results provide helpful information for the mental health counseling and intervention of freshmen’s mental problems.

## Conclusion and discussion

In this research, we have taken PPS (probability proportionate to size sampling) investigations about the mental problems to thousands of freshmen in 45 public universities in Shandong Province near the end of their first semester. Based on these data, the comparison of mental problems for post pandemic era vs. pre pandemic era and the influencing factors for anxiety and depression were analysed. From the survey and analysis, the extents of anxiety and depression in post pandemic era are significantly more severe than pre pandemic era, which confirms the viewpoints in former research, such as^32^ and^33^. Inferiority complex, obsession and somatization are the main mental effect factors of anxiety and depression. As the first systematical investigation and analysis for the mental problems among freshmen in the whole Shandong Province before and after the epidemic, the results in this manuscript substantially extend former research in this area. Based on these analysis results, the psychological counseling institutions can provide more targeted psychological health education and psychological intervention for the freshmen with anxiety and depression, and offer adequate and appropriate psychological support services for them.

### Strengths and limitations

To the best of our knowledge, this research is the first systematical investigation and analysis for the mental problems among freshmen in the whole Shandong Province before and after the epidemic. In addition, our sampling investigation adopted PPS sampling, and reasonably determined the sample size, which guaranteed the representative of the sample to the statistical population.

There are also some limitations to this study. First, the status of mental problems was based on the freshmen’s self-assessment questionnaire rather than clinical diagnoses, thus reporting bias may exist when compared with the professional assessment. Second, this survey mainly used Chinese college students’ mental health screening scale, which focused on the psychological problems of college students. Some other effect factors for mental problems may not be included in this scale.

Future works include but not limited to the experimental design for treatments of anxiety and depression by physical activities and other measures. These are interesting and challenging works, which will be researched in the near future.

## Data Availability

The data sets which support this study are available from the corresponding author upon reasonable request.

## References

[1] Berney, T., Black, S., Checinski, K., Crome, I., Feinmann, C., Gowers, S., Hobbs, M., Marsden, A., Mahmood, T. & Smith, E. The Mental Health of Students in Higher Education, Royal College of Psychiatrists.

[2] Stewart-Brown S, Evans J, Patterson J, Petersen S, Doll H, Balding J, Regis D (2000). The health of students in institutes of higher education: An important and neglected public health problem?. J. Public Health Med..

[3] Tomoda A, Mori K, Kimura M, Takahashi T, Kitamura T (2010). One-year prevalence and incidence of depression among first-year university students in Japan: A preliminary study. Psychiatry Clin. Neurosci..

[4] Adlaf EM, Gliksman L, Demers A, Newton-Taylor B (1998). The prevalence of elevated psychological distress among Canadian undergraduates: Findings from the 1998 Canadian Campus Survey. J. Am. Coll. Health.

[5] Wong, J. G., Cheung, E. P., Chan, K. K., Ma, K. K. & Wa Tang, S. Web-based survey of depression, anxiety and stress in first-year tertiary education students in Hong Kong. *Aust. N. Z. J. Psychiatry* (9). 10.1080/j.1440-1614.2006.01883.x 16911753

[6] Cheung, K., Tam, K. Y., Tsang, M. H., Zhang, L. W. & Lit, S. W. Depression, anxiety and stress in different subgroups of first-year university students from 4-year cohort data. *J. Affect. Disord.* **274**. 10.1016/j.jad.2020.05.041 32469820

[7] Wei L (2015). Prevalence and related risk factors of anxiety and depression among Chinese college freshmen. J. Huazhong Univ. Sci. Technol..

[8] Bao Y, Sun Y, Meng S, Shi J, Lu L (2020). 2019-nCoV epidemic: Address mental health care to empower society. Lancet.

[9] Shi, L., Lu, Z. A., Que, J. Y., Huang, X. L., Liu, L., Ran, M. S., Gong, Y. M., Yuan, K., Yan, W., Sun, Y. K. & Shi, J. Prevalence of and risk factors associated with mental health symptoms among the general population in China during the coronavirus disease 2019 pandemic. *JAMA Netw. Open*. 10.1001/jamanetworkopen.2020.14053 PMC7330717 32609353

[10] Vadivel R, Shoib S, Halabi SE (2021). Mental health in the post-COVID-19 era: Challenges and the way forward. Compr. Psychiatry.

[11] Osaghae, I., Nguyen, L. K., Tong, H. C., Moffitt, O. & Hwang, K. O. Prevalence and factors associated with mental health symptoms in adults undergoing Covid-19 testing. *J. Prim. Care Commun. Health* **12**. 10.1177/21501327211027100 PMC8246585 34184942

[12] Zhou SJ, Wang LL, Qi M, Yang XJ, Chen JX (2021). Depression, anxiety, and suicidal ideation in Chinese university students during the COVID-19 pandemic. Front. Psychol..

[13] Xiong, J., Lipsitz, O., Nasri, F., Lui, L. & Mcintyre, R. S. Impact of COVID-19 pandemic on mental health in the general population: A systematic review. *J. Affect. Disord.* 10.1016/j.jad.2020.08.001 PMC7413844 32799105

[14] Zheng XE (2021). A cross-sectional study on mental health problems of medical and nonmedical students in Shandong during the COVID-19 epidemic recovery period. Front. Psychiatry.

[15] Su R, Chen X, Zhao M (2021). A preliminary study on the mental health status and influencing factors of college students during the epidemic of New Coronary Pneumonia—taking Shandong University of Traditional Chinese Medicine as an example. Health Vocat. Educ..

[16] Zhang W (2021). Research on the anxiety of college graduates under the COVID-19 pandemic. West. Qual. Educ..

[17] Fang X, Yuan X, Wei HU (2018). The development of college students mental health screening scale. Stud. Psychol. Behav..

[18] Baker R, Brick JM, Bates NA, Battaglia M, Couper MP, Dever JA, Gile KJ, Tourangeau R (2013). Summary report of the AAPOR task force on non-probability sampling. Hisp. J. Behav. Sci..

[19] Tourangeau R, Conrad FG, Couper MP (2013). The Science of Web Surveys.

[20] Winiowski A, Sakshaug JW, Ruiz D, Blom AG (2020). Integrating probability and nonprobability samples for survey inference. J. Surv. Stat. Methodol..

[21] Triola MF (2017). Elementary Statistics.

[22] Zhu, T., Jian, L. I. & Zhang, S. S. Status and influencing factors of anxiety and depression among freshmen in North Sichuan Medical College, Occupation and Health.

[23] Liu, J.-J., Gao, Y. X. & Zhang, T. N. University, Research on current status and influential factors of anxiety and depression among freshmen in a college. *Jiangsu J. Prevent. Med.*

[24] Choi, K. W., Kim, Y. K. & Hong, J. J. Comorbid anxiety and depression: Clinical and conceptual consideration and transdiagnostic treatment. 10.1007/978-981-32-9705-0_14 32002932

[25] Khansa, W., Haddad, C., Hallit, R., Akel, M., Obeid, S., Haddad, G., Soufia, M., Kheir, N., Hallit, C. A. E. & Khoury, R. A. Interaction between anxiety and depression on suicidal ideation, quality of life, and work productivity impairment: Results from a representative sample of the Lebanese population. *Perspect. Psychiatric Care* **56**. 10.1111/ppc.12423 31321788

[26] Liu, S., Gong, D., Hua, Z. & Wu, M. Research II: On Armymen’s emotions of anxiety and depression: Leading factors and their interaction. *Psychol. Sci.*

[27] Roy-Byrne PP (1996). Generalized anxiety and mixed anxiety-depression: Association with disability and health care utilization. J. Clin. Psychiatry.

[28] Kessler RC, Berglund P, Demler O, Jin R, Koretz D, Merikangas KR, Rush AJ, Walters EE, Wang PS (2003). The epidemiology of major depressive disorder: Results from the National Comorbidity Survey Replication. JAMA J. Am. Med. Assoc..

[29] Cameron OG (2006). Anxious-depressive comorbidity: Effects on HPA axis and CNS noradrenergic functions. Essent. Psychopharmacol..

[30] Lamers, F., van Oppen, P., Comijs, H. C. & Johannes, H. Comorbidity patterns of anxiety and depressive disorders in a large cohort study. *J. Clin. Psychiatry*. 10.4088/JCP.10m06176blu 21294994

[31] Xu, Z. Martin Bland (2015): An introduction to medical statistics. Statistical Papers.

[32] Chen C, Zhou H, Wei Y (2021). Research on psychological status and follow-up intervention of anxiety disorder patients under the COVID-19 epidemic. Mod. Med. Health.

[33] Lai Y, Zhang H, Wu Y (2022). The influence of daily behaviors of medical students in a closed management university on their mental health. Psychol. Prog..

[34] Fei Y (2014). Multivariate Statistical Analysis with R.

